# Uterine Metabolomic Analysis for the Regulation of Eggshell Calcification in Chickens

**DOI:** 10.3390/metabo11090575

**Published:** 2021-08-26

**Authors:** Xiqiong Wang, Ping Zhu, Zhihua Sun, Junnan Zhang, Congjiao Sun

**Affiliations:** 1 National Engineering Laboratory for Animal Breeding and Key Laboratory of Animal Genetics, Breeding and Reproduction, Ministry of Agriculture and Rural Affairs, College of Animal Science and Technology, China Agricultural University, Beijing 100193, China; xqwang@cau.edu.cn (X.W.); zhupingcau@163.com (P.Z.); cauzhangjn@163.com (J.Z.); 2 National Animal Husbandry Service, Ministry of Agriculture and Rural Affairs, Beijing 100125, China; goodluck_szh@163.com

**Keywords:** chicken, uterine fluid, eggshell quality, metabolomics, metabolites

## Abstract

Eggshell quality is economically important for table eggs and functionally indispensable for hatching eggs. During the formation of eggshell in the uterus, organic matrixes in uterine fluid can control and modify the formation of calcified eggshell. At present, there are limited studies focusing on the effect of uterine organic metabolites on eggshell quality. In this study, an LC-MS-based metabolomic technology was performed to identify the crucial uterine metabolites that differently presented in hens producing eggs with divergent eggshell quality (eggshell strength, thickness, and weight). More than 1000 metabolites were identified in uterine fluid, and six putative metabolites, including phosphatidylcholine, diacylglycerol, verapamil, risedronate, coproporphyrinogen III, and biliverdin, were screened to play crucial roles in eggshell calcification. Then, two trials for oral administration and in vitro calcite crystal growth were conducted to verify the effect of potential different metabolites on the eggshell quality. Verapamil has a temporary effect on decreasing eggshell strength and eggshell thickness. Coproporphyrinogen III could induce smaller calcite crystals to improve eggshell strength while biliverdin could modify crystal morphology by forming rougher faces and rounder edges to strengthen the eggshell. The present study gives us new insight to understand the role of uterine fluid matrixes in eggshell calcification.

## 1. Introduction

Eggshells perfectly separate the internal contents of the egg from the external environment and provide a natural barrier, aiding the storage of table eggs and the development of embryos. Eggshells can guarantee the physical integrity of the egg and protect the egg from contamination with microorganisms. Numerous pores on the surface of eggshells act as gas exchange channels during the growth of avian embryos, and the eggshell provides a calcium source for developing embryos [1,2]. Most notably, eggshell quality plays a crucial economic role in egg production, accounting for 8~10% of total egg production losses from eggshell breakage, causing economic harm [3]. Therefore, it is economically important to improve eggshell quality for the production of eggs for consumption and hatching.

It takes a fairly long time, approximately 18~19 h, to form a highly organized and calcific eggshell with a special bioceramic structure during the entire egg forming cycle. As the developing egg reaches the isthmus and uterus five hours after ovulation, the dilated uterus secretes large quantities of calcium and bicarbonate as well as small amounts of proteins and other chemicals into the uterine fluid [3,4,5]. These secretions form highly organized deposits constituting the main components of eggshells. In general, eggshells are composed of 95% inorganic minerals, 3.3–3.5% organic matrix, and 1.6% water. Additionally, calcium carbonate is the main component of the eggshell and accounts for approximately 94% [6]. According to the deposition order and morphology of these materials, the eggshell is divided into six visible layers from the inside out: the inner shell membrane, outer shell membrane, mammillary layer, palisade layer, vertical crystal layer, and cuticle [6]. Similarly, the whole calcification process is known to consist of three stages [1,7], i.e., the initiation of mineralization (lasting approximately 5 h), the growth phase (taking approximately 12 h), and the termination of calcification (lasting approximately 1.5 h). In particular, the composition of the uterine fluid varies with different stages of eggshell formation and influences the morphology of calcite in the different zones of the eggshell [1].

Recently, how organic matrixes change in the uterine fluid during eggshell calcification and how these organic matrixes affect eggshell quality have attracted increasing attention. Gautron et al. (1997) observed the precursors of the eggshell matrix present in the uterine fluid and involved in the process of eggshell calcification [8]. Marine et al. (2015) identified different dominant proteins present during different stages of shell calcification and found that ovocleidin-17 (OC-17) is mainly present at the initiation of calcification [9]. Sun et al. (2013) reported that several eggshell matrix, calcium binding and transport, bone development, or disease-related proteins are closely associated with strong eggshell formation [10]. Certain proteins, such as osteopontin, ovalbumin, lysozyme, ovotransferrin -, OC-17, and ovocleidin-116, have been demonstrated to influence calcite crystal morphology in vitro [11,12,13,14,15,16,17,18] and further affect eggshell quality or mechanical properties. However, with the exception of eggshell matrix proteins, it remains unknown whether other organic substances in the uterine fluid, such as the end products of cellular processes—metabolites—function in eggshell calcification, calcite crystal growth, and eggshell quality.

Metabolomics makes it possible to systematically and comprehensively identify the changes in cellular or organismal metabolites, offering a direct readout of biochemical activity that can result from genetic perturbation or environmental changes. Thus, in this study, we aimed to investigate the differential metabolites in uterine fluids collected from hens with divergent eggshell quality and to decipher the precise roles of these metabolites in eggshell quality through verification assays, enabling us to gain new insight into the crucial functions of these metabolites in eggshell quality and calcification.

## 2. Results

### 2.1. Comparisons of Eggshell Quality Traits

In general, a thicker or heavier eggshell often means a stronger egg. However, in some cases, eggshell strength varies greatly even if these eggs have similar eggshell thickness and weight. Hence, three groups of hens (6 for each group) were selected from a population of 183 individual chickens. The first group of hens produced eggs with strong, thick, and heavy eggshells (STH). To emphasize the eggshell strength trait, the second group of hens produced eggs with thick and heavy but weak eggshells (WTH). The third group of hens produced eggs with thin, light, and weak eggshells (WtL). Table 1 shows the comparisons of eggshell thickness, weight, and strength among these three groups. Compared to the WTH group, the STH group had a 1.67-fold higher shell strength (3.53 vs. 2.11 kg/cm^2^, *p* < 0.05) but equivalent shell thickness (0.327 vs. 0.314 mm) and weight (6.12 vs. 5.78 g, *p* > 0.05), while the WtL group had thinner shell thickness (0.257 vs. 0.314 mm, *p* < 0.05) and lighter eggshell weight (4.73 vs. 5.78 g, *p* < 0.05) but similar eggshell strength (1.69 vs. 2.11 kg/cm^2^, *p* > 0.05). The comparison of uterine metabolites among these three groups could reveal the key molecules that regulate eggshell formation and strength. In addition, the eggshells of these three groups had similar egg weights and shape indices (*p* > 0.05), which could minimize the error.

### 2.2. Metabolite Profiles of Uterine Fluid

**Figure 1:**
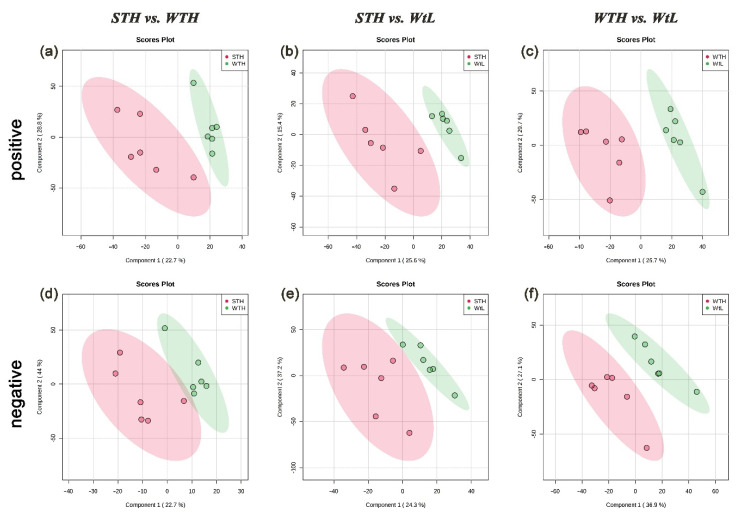
Partial least squares-discriminant analysis (PLS-DA) model of uterine fluid metabolites. (**a**–**c**) PLS-DA score plots of uterine fluid samples collected from hens with STH, WTH, and WtL eggshells in positive ion mode; (**d**–**f**) PLS-DA score plots of samples collected with negative ion mode. Each point in the graph represents one sample, and the discretization of the two-color symbols represents the distribution of the two sets of samples on the PC1 and PC2 axes. STH, hens with strong, thick, and heavy eggshells; WTH, hens with weak, thick, and heavy eggshells; and WtL, hens with weak, thin, and light eggshells.

**Figure 2:**
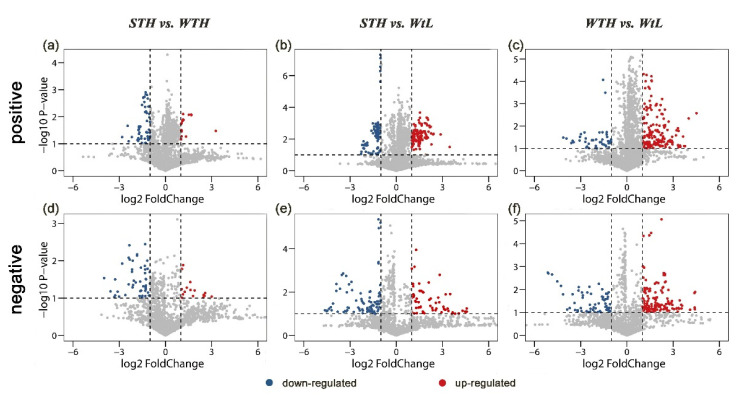
Volcano plots of uterine fluid collected from the STH, WTH, and WtL groups in the positive (**a**–**c**) and negative (**d**–**f**) ion modes. Red dots represent significantly upregulated metabolites, and blue dots represent significantly downregulated metabolites in the comparison group (*p* < 0.05 and fold change ≥ 2). STH, hens with strong, thick, and heavy eggshells; WTH, hens with weak, thick, and heavy eggshells; and WtL, hens with weak, thin, and light eggshells.

During the rapid growth phase of avian eggshell calcification, the type and quantity of organic and inorganic components in the uterine fluid affect the precipitation of calcium carbonate and further affect eggshell quality. To understand the role or function of small molecule metabolites in eggshell calcification, Liquid chromatography–mass spectrometry (LC–MS) was performed to detect these small molecule metabolites in hen uterine fluid in both positive and negative modes. More than 1000 metabolites were identified in the uterine fluid of the 3 groups of hens. Then, the results of Partial least squares-discriminant analysis (PLS-DA) showed strong separations (Figure 1), i.e., the samples in ellipsoids are obviously far away from each other, indicating that the uterine fluid samples had strong repeatability and consistency in the same group and great differences in the divergent group. Volcano plots suggested that 21 and 57 metabolites were significantly upregulated in the STH and WTH groups, respectively, in positive mode (Figure 2a). When the metabolites of uterine fluids in the STH group were compared with those in the WtL group, 125 metabolites were significantly upregulated, while 90 metabolites were obviously downregulated (Figure 2b). There was also a significant difference between the WTH group with 168 overrepresented metabolites and the WtL group, with 44 metabolites being highly represented (Figure 2c). In addition, in negative ion mode, the three groups also exhibited visible divergence (Figure 2d–f).

### 2.3. Identification of Differentially Present Metabolites

The metabolites detected in both the positive and negative ion modes were used for the differential analysis according to the criteria of a fold change > 2 and *p*-value < 0.05. After matching their names with those in the PubChem database, 23 differential metabolites were identified between the STH and WTH groups (see Table S1), 42 differential metabolites between the STH group and WtL group (see Table S2), and 52 differential metabolites between the WTH and WtL groups (see Table S3). Then, the related metabolic pathways were further searched in the Kyoto Encyclopedia of Genes and Genomes (KEGG) pathway, and only several differential metabolites identified in the positive ion mode were enriched in the glycerophospholipid metabolism and linoleic acid metabolism pathways (Table 2).

### 2.4. Putative Metabolites Potentially Participating in Eggshell Calcification

According to the results of the KEGG pathway analysis and the literature search, six metabolites were screened as candidate molecules affecting eggshell calcification and quality (Table 3). In both positive and negative modes, phosphatidylcholine (PC), involved in glycerophospholipid metabolism and alpha-linoleic acid metabolism, was detected. In positive ion mode, diacylglycerol (DG), which primarily functions to activate protein kinase C (PKC), was identified, and two eggshell pigment molecules, biliverdin and coproporphyrinogen III (CpgenIII), associated with eggshell mineralization, were observed. The concentrations of biliverdin and CpgenIII in the STH group were 3.088 and 2.656 times higher than those in the WtL group, respectively. This suggests a potential relationship between these two pigment molecules and eggshell quality. In addition, verapamil and risedronate were detected in negative ion mode, and both of them had higher concentrations in the WTH uterine fluid, indicating that verapamil facilitated the formation of thick and heavy eggshells, while risedronate could prevent eggshell strengthening.

### 2.5. Verification of the Effects of Verapamil and Risedronate on Eggshell Quality

**Figure 3:**
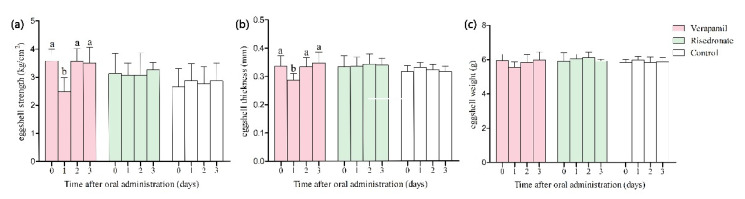
Effect of oral administration of verapamil and risedronate on eggshell quality, including (**a**) strength, (**b**) thickness, and (**c**) weight. The White Leghorn hens were fed verapamil hydrochloride (8 mg/hen), sodium risedronate (1.75 mg/hen), and empty capsules, respectively. Eggs were collected on the day capsules were fed and on the successive 3 days after oral administration, and then eggshell quality was measured. Data are represented as means ± SEM (n = 10 hens per group). Statistical significance of mean differences was tested by one-way ANOVA. *p* < 0.05 (a,b).

Both verapamil and risedronate had higher concentrations in the WTH group than in the WtL and STH groups, respectively. The metabolites verapamil and risedronate cannot be synthesized in chickens and can only be gained through feed intake or injection. It is hypothesized that exogenous verapamil and risedronate could decrease eggshell strength, thickness, and weight. To confirm this hypothesis, these two metabolites were fed to hens by capsules, and then the three eggshell quality traits of the subsequently produced eggs were measured. The results showed that on the first day after feeding, hens fed verapamil produced eggs with a significantly lower eggshell strength (decreased by 30.4%, *p* < 0.05) and thickness (decreased by 14.3%, *p* < 0.05) (Figure 3). From the second day, both eggshell strength and thickness recovered to their levels on the feeding day, indicating that verapamil is likely to be mobile in hens’ bodies for only a short time and has a temporary effect, decreasing eggshell strength and thickness. Therefore, it could be speculated that verapamil maintains higher levels in the uterine fluid of WTH hens due to its larger number of residues. Additionally, there were no significant differences in the eggshell strength, thickness, or weight of eggs produced by hens fed risedronate capsules or empty capsules (control group). Hence, risedronate has no effect on eggshell quality under normal rearing conditions.

### 2.6. Verification of the Effects of cpgenIII and Biliverdin on Calcite Crystal Morphology

**Figure 4:**
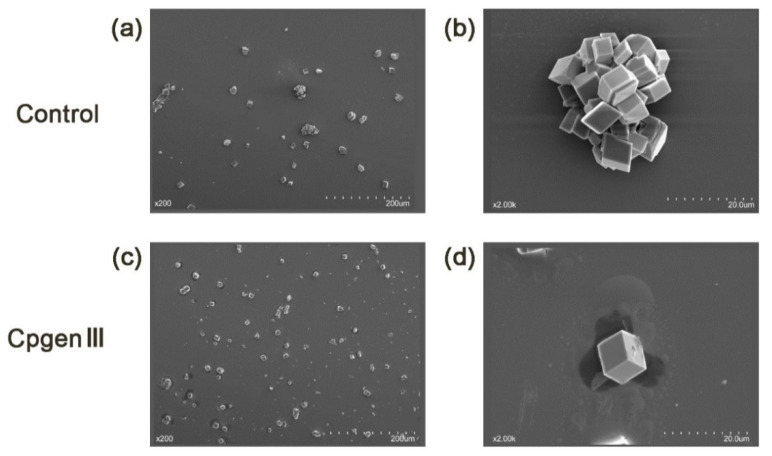
Scanning electron microscopic view of calcite crystals grown in the absence or presence (**a**,**b**, control) of CpgenⅢ (**c**,**d**). The scale bars are indicated at the bottom right of the pictures, and the magnification is shown at the bottom left.

**Figure 5:**
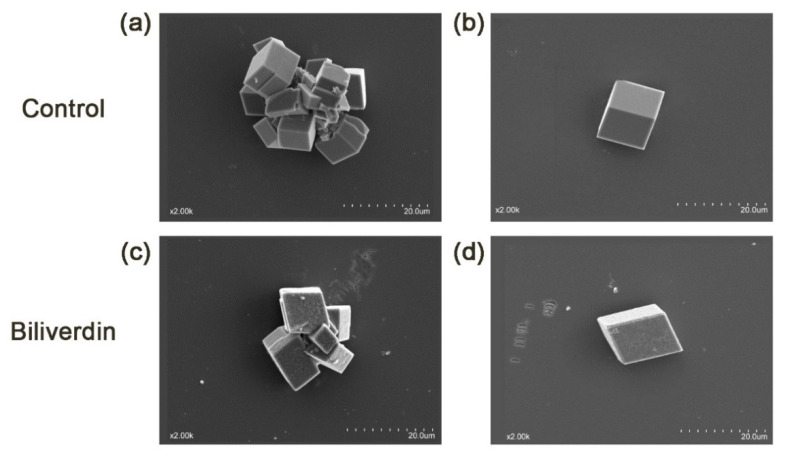
Scanning electron microscopic view of calcite crystals grown in the absence or presence (**a**,**b**, control) of biliverdin (**c**,**d**). (**a**,**c**) View of aggregated crystals of the blank and biliverdin-treated groups, respectively. (**b**,**d**) View of single-separated crystals of the blank and biliverdin-treated groups, respectively. The scale bars are indicated at the bottom right of the pictures, and the magnification is shown at the bottom left.

Two major eggshell pigments, cpgenIII (the precursor of protoporphyrin IX (PPIX)) and biliverdin, had a higher content in the STH group than in the WtL group. Given that certain components in the uterine fluid, especially eggshell matrix proteins [11,12,13,14,15,16,17,18], change the calcite morphology and growth rate in different ways, it was hypothesized that these two pigments may also have affected the calcite structure. Hence, an in vitro crystallization assay was conducted to study whether these two pigments affect calcite crystal morphology. In the control group (containing only CaCl_2_ solution), all calcite crystals with a size of 10 to 20 μm displayed a characteristic cleaved <104> rhombohedra. Both the aggregated and single calcites displayed sharp edges and smooth faces, as shown in Figure 4a,b and Figure 5a,b. Then, CpgenIII and biliverdin were added to a constant concentration of calcium chloride (CaCl_2_) solution to observe the change in calcite precipitation, especially as is it related to crystal morphology. The addition of CpgenIII resulted in rhombohedral <104> faces, but the size of the calcite crystals decreased by approximately 10 μm (Figure 4c,d). Most of the calcite existed as single separated crystals rather than aggregated crystals. The introduction of biliverdin resulted in crystals with the same size but clearly different morphology compared to the control. The faces became rough and uneven, and the corners became rounder. Moreover, all the edges of the rhombohedra became inclined, not vertical or horizontal (Figure 5c,d).

## 3. Discussion

Eggshell calcification takes place in the uterus of hens. There is no doubt that the components of uterine fluid provide the dominant sources of eggshell matrix and play an important role in the formation of eggshell. Although the uterine fluid components of hens, especially matrix proteins, have been universally studied, the precise composition and crucial metabolites in the uterine fluid relating to eggshell formation remain unknown. These metabolites are likely to regulate the deposition and metabolization of calcium carbonate and then further affect eggshell quality. The current study was therefore carried out with a divergent eggshell quality model, and a metabolomics approach based on LC-MS was used to detect the differential metabolites in the uterine fluid of hens with eggs with divergent eggshell quality.

The eggshells in the STH group had a higher strength than those in the WTH group, which was probably caused by the different types and amounts of small molecule metabolites in the uterine fluid. When the WTH group was compared to the WtL group, the eggshell strength and eggshell weight differed significantly, possibly due to the deposition of calcium carbonate. The STH group and the WtL group had obvious differences in eggshell strength, thickness, and weight, which was likely due to the comprehensive effect of calcium carbonate deposition, small molecule type, and amount. The hens used in our experiments were under the same conditions but had divergent eggshell quality, so detecting the differential metabolites in the uterine fluid of these three groups of hens could be of significance to screen some potential small molecules that affect eggshell quality.

Our results showed that the PC and DG contents in the STH group were significantly higher than those in both the WTH and WtL groups. In fact, the relationship between PC and eggshell strength was clearly demonstrated late in the last century. Castaldo and Maurice (1988, 1989, 1997) observed that hens with strong shells tend to have a greater concentration of PC in their uterine fluid and a higher calcium adenosine triphosphatase (Ca^2+^-ATPase) activity in their uterine mucosa than hens producing weak shells, which agrees with our results and confirms that PC is crucial to eggshell calcification and may exert its role by altering the Ca^2+^-ATPase activity of the uterine fluid [19,20,21].

It is well known that DG functions primarily in activating PKC, and sustained generation of DG could easily achieve the postulated prolongation of PKC activation [22]. The PKC family, consisting of 10 structurally related serine/threonine protein kinases [23], plays an important role in the process of phosphorylation in cells of a range of organisms. In chickens, some eggshell matrix proteins are phosphoproteins that are considered to control and regulate calcium carbonate precipitation during eggshell calcification, mainly including osteopontin and the eggshell-specific proteins ovocleidin-17, ovocleidin-116, and ovocalyxin-32 [24]. Osteopontin has been revealed to be associated with eggshell thickness [25] and fracture toughness [26] and to be expressed at a reduced or absent level in specific regions of the uterine epithelium of hens producing pimpled or corrugated shells [27]. From the perspective of the eggshell microstructure, uterine osteopontin could guide calcite orientation and alter crystal morphology by inhibiting calcite growth at characteristic rhombohedral <104> faces [11]. Dephosphorylation of the serine residues of osteopontin abolishes its ability to inhibit calcium carbonate precipitation [28]; therefore, it can be speculated that phosphorylation of osteopontin enables its ability to modify calcite crystals and then affect eggshell quality. In addition, phosphate-induced osteopontin expression specifically requires a PKC-dependent signaling pathway [29]. Hence, it is highly likely that high levels of DG ensure the sustained activation of PKC and further facilitate the functioning of osteopontin. Furthermore, there is no evidence that other phosphorylated eggshell matrix proteins require the participation of the PKC signaling pathway, and the case of osteopontin suggests that this possibility should be further explored.

In our study, verapamil and risedronate were detected in the uterine fluid. It is clear that hens are unable to synthesize these two chemicals on their own, which can only be deposited into hens’ bodies through dietary supplementation or medicine injection. In the poultry industry, enrofloxacin is applied most frequently to treat colibacillosis, a common disease in poultry. Oral administration of verapamil could significantly improve the absorption of enrofloxacin in both healthy and infected broilers [30]. In addition, it is noted that verapamil is a well-known calcium channel blocker. In our study, verapamil was found to have a higher content in the WTH group than in the WtL group, suggesting that verapamil may play a positive role in regulating the deposition of calcium carbonate. In contrast, during oral verapamil treatment, the eggshell strength and thickness obviously declined first and then recovered to the same level as that at the day of feeding. Verapamil can inhibit calcium influx through the slow channels of vascular smooth muscle and decrease calcium contents in the plasma of rats [31]. Therefore, in chickens, it is hypothesized that a high dose of verapamil will reduce the level of plasma calcium. Since calcium in uterine fluid is supplied continuously by blood plasma via transepithelial transport during eggshell formation [32,33], calcium in uterine fluid is reduced, and the deposition of calcium carbonate in eggshell further decreases. This approach resulted in a decrease in eggshell thickness after verapamil capsules were fed to hens. Kibala et al. (2015) found a genetic correlation between eggshell strength and thickness of approximately 0.8 [34]; therefore, a decrease in eggshell thickness after oral verapamil administration was related to a decline in eggshell strength. Notably, eggshell thickness and strength rapidly returned to normal, indicating that verapamil is metabolized in a short time. Early studies focused on the effects of verapamil on chicken biventer-cervicis muscle [35], atherosclerotic chickens [36,37], and shifts in drug–drug interactions when broilers were treated with the specific P-glycoprotein inhibitor verapamil [38]. It is uncommon to add verapamil to the chicken’s diet during egg production, and few studies have focused on the effects of verapamil on layers and chicken egg quality although verapamil could attenuate lipopolysaccharide [39] and colibacillosis [30] in broilers when treated with oral enrofloxacin. Hence, our results first showed verapamil has a temporary effect on declining eggshell thickness and strength. It is unknown whether verapamil could be deposited in egg yolk or egg white, which needs further study.

In commercial caged egg-laying hens, approximately 20% of hens suffer from bone breakage and mortality, with osteoporosis accounting for up to one-third of the total mortality [40,41]. Risedronate, a pyridinyl bisphosphonate, has been reported to be an ideal therapy option in the prevention and treatment of osteoporosis in humans [42,43,44,45] and to improve bone architecture and quality in rats [46]. Hence, in some farms, ridedronate dietary supplementation is likely to be considered to improve bone breaking strength and relieve osteoporosis in laying hens, whereas it is unclear whether risedronate could accumulate in the egg content. In the current study, we found that the concentration of risedronate in the uterine fluid of WTH hens with poor eggshell quality was greater than that of STH hens with good eggshell quality. Therefore, risedronate may have some impact on eggshell strength. Oral administration to White Leghorn chickens resulted in no differences in the eggshell strength, thickness, or weight of eggs produced by hens fed 1.75 mg of risedronate, suggesting that risedronate is not the main factor that affects eggshell strength.

Interestingly, eggshell pigments have a higher concentration in the uterine fluid of relatively strong, thick, and heavy eggshell. The eggshell is mainly composed of calcite, a colorless and transparent crystal under natural conditions. However, in the last 5 h before oviposition, approximately 50% to 74% of pigments are deposited on shells [47], so eggshells are usually colored white, brown, blue, or other colors. Large amounts of PPIX and trace amounts of biliverdin IX are the predominant components of eggshell pigments [48,49,50] and are synthesized in the uterus of the hen’s oviduct [51,52,53,54]. In this study, it was observed that in the uterus, the STH group had higher contents of biliverdin and cpgenIII than the WtL group, indicating that these two pigments may play a key role in improving eggshell quality. However, for chickens, several studies have been more focused on the relationships between eggshell darkness and eggshell quality, not eggshell pigment and eggshell quality. Godfery (1949) observed that darker brown eggs have a higher eggshell strength and thickness than lighter brown eggs, and eggshell color positively correlated with eggshell quality [55]. Later, Aygun (2014) similarly concluded that eggshell strength increased as the darkness of eggshells increased [56], and Sekeroglu et al. (2011) showed that the shell color of brown eggs is significantly affected by eggshell thickness [57]. It has been proposed that eggshell color could be used as an indicator to assess some eggshell quality parameters, such as strength, thickness, and weight, according to their close correlation [58,59], although some conflicting evidence exists. Since eggshell color is determined by pigment concentrations and types, all of these studies suggested that eggshell pigments can affect shell quality. Fortunately, other studies using wild avian eggs to explore relationships between eggshell pigment and eggshell quality can support this theory.

CpgenIII is transported into the mitochondrial intermembrane space and then is converted to PPIX by coproporphyrinogen oxidase and protoporphyrinogen oxidase [60]. The structure of PPIX is close to that of phthalocyanines, solid-state lubricants used in engineering, so it has been proposed that PPIX may function as a lubricant in the shell matrix and may improve eggshell strength [61,62]. For speckled eggs, protoporphyrin is reported to compensate for reduced eggshell thickness, with spots specifically demarcating thinner areas of shell [63,64]. Gosler et al. (2011) confirmed that protoporphyrin serves as a substitute for calcium to strengthen thinner areas of eggshell and has a localized strengthening effect on the eggshell’s toughness, which might be achieved by a shock absorption effect within the eggshell [65]. According to the abovementioned results, it is hypothesized that for brown eggs, a greater amount of cpgenIII is eventually converted into more PPIX on the eggshell with a relatively uniform distribution during the last 5 h after oviposition, so the eggshell thickness is increased followed by reinforcement of the eggshell. However, the relationship of another pigment, biliverdin, with eggshell quality remains controversial. Jagannath et al. (2008) showed that biliverdin, which produces a bluish-green color, has no significant association with eggshell thickness [64]. Butler and Waiter (2016) reported that biliverdin concentration increased with decreased eggshell thickness using European starling (*Sturnus vulgaris*) eggs [66]. Hence, whether biliverdin plays an important biomechanical role remains to be further studied.

Because eggshell microstructure (including the size, shape, and orientation of crystals) influences eggshell mechanical properties and the quantity and composition of shell matrix components are associated with changes in the microstructure, it is hypothesized that cpgenIII and biliverdin could influence the modification of calcite crystals. In the in vitro assay of calcite crystal growth, the control group exhibited characteristic rhombohedral phases <104>, indicating that the methods were reliable and that the results were accurate. Crystal morphology is the consequence of the interaction between specific calcite crystal faces and biological molecules [67]. In the current study, the precise roles of pigments in eggshell microstructure can therefore be inferred according to the mediated morphological modifications of the rhombohedric crystals; a smaller crystal size and lower heterogeneity of crystal size were caused by cpgenIII additives. Previous studies have reported that chicken eggshells are much stronger and consist of smaller crystals [68,69], and our study has shown that the content of cpgenIII in the STH group was more than three times that in the WtL group. Therefore, cpgenIII could improve eggshell strength by forming smaller crystals. This inference can be clarified by the increased boundary areas of the crystals as the crystal size decreases, making it more difficult for eggs to crack [70]. In addition, biliverdin additives resulted in rough and uneven rhombohedral <104> crystals. Similarly, biliverdin had a higher content in the STH group, so it is highly likely that biliverdin could make calcite crystal faces rougher to strengthen eggshells. It is not surprising that the rougher the material surface is, the slower cracks spread [71], i.e., the roughness of the material surface inhibits the spread of cracks. Thus, a crack takes more external energy to spread through rough and uneven crystals than between smoother crystals. Overall, by inducing smaller or rougher calcite crystals, higher concentrations of cpgenIII and biliverdin in the uterine fluid could enhance the eggshell mechanical properties.

## 4. Materials and Methods

### 4.1. Bird Management

In this experiment, 200 340-day-old White Leghorn hens kept on our experimental farm at China Agricultural University were used. All birds were caged individually and were subjected to a 16-h light and 8-h dark cycle (16L: 8D). These birds were fed as recommended by the National Poultry Performance Test Center (Table 4) and the whole experimental procedure was strictly performed according to the protocol approved by the Animal Welfare Committee of China Agricultural University (AW62801202-1-1).

### 4.2. Measurements of Egg Traits and Selection of Hens

Fresh eggs were collected from 183 hens of experiment flock (91.5% of laying hens) for 3 successive days, and egg quality traits were measured on the day of sampling. Egg weight was measured using an electronic scale with an accuracy of 0.1 g. The length and width of eggs were measured with an Egg Shape Index Gauge (FHK, Tokyo, Japan, 1 mm; shape = length/width). Then eggshell strength was measured vertically with the blunt end of each egg up using the Eggshell Force Gauge (Model-II, Robotmation, Tokyo, Japan). Subsequently, each egg was cracked, the eggshell with membrane was dried with paper towel to remove residual egg white, and then it was weighed using an electronic scale. After, eggshell thickness was calculated averagely by the thickness of the blunt, middle, and sharp end of the eggshell without membrane and measured using a Coolant Proof Micrometer (Mitutoyo, Kawasaki, Japan). Finally, 18 hens were eventually selected and divided into 3 groups (STH, hens with strong, thick, and heavy eggshell; WTH, hens with weak, thick, and heavy eggshell; WtL, hens with weak, thin, and light eggshell) for their divergent eggshell weight, eggshell strength, and eggshell thickness (Table 1).

### 4.3. Uterine Fluid Sampling

The selected 18 hens were kept individually in adjacent cages and were observed for several days from 5:00 am to 6:00 pm to record the precise laying time of each hen. Therefore, we were able to precisely predict the ovulation time within a laying sequence for each hen. During the phase of eggshell mineralization in hens 18 h after previous oviposition, uterine fluid was collected after the hen was injected with 0.3 mL of cloprostenol (0.16 mg/mL; Dandong Ludan Hehua Animal Pharmaceutical Co., Ltd., Dandong, China) in the wing vein, which induced oviposition with concurrent uterine fluid loss from the oviduct. Then, a plastic tube was placed at the entrance of the everted vagina immediately after the egg was produced. Uterine fluid was diluted in Tris-HCl buffer (0.0625 M, pH 6.8, the volume ratio of sample to buffer was 1:1, 10 mM Roche protein inhibitor-Aprotinin was included), and then rapidly frozen in liquid nitrogen to limit any spontaneous precipitation of calcium carbonate and proteins and finally stored at −80 °C for the subsequent liquid chromatography–mass spectrometry (LC–MS) analysis.

### 4.4. Sample Preparation for MS Analysis

Prior to analysis, 300 μL of acetonitrile were added to 100 μL of each uterine fluid sample in a clean 1.5-mL Eppendorf tube. The mixtures were vortexed vigorously for 1~3 min, placed at 4 °C for 10 min, and then centrifuged at 13,000 rpm for 10 min. The supernatant was transferred into a new 1.5-mL Eppendorf tube and dried in a vacuum dryer. Then, the dried samples were reconstituted with 100 μL of acetonitrile for further analysis.

### 4.5. LC-MS Analysis

LC-MS metabolomics analysis was performed on an ultra-high-performance liquid chromatograph (Nexera X2 system, Shimadzu, Kyoto, Japan) and high-resolution mass spectrometer (Triple TOF 5600+, AB SCIEX, Redwood City, CA USA) equipped with a dual electrospray ionization (ESI) source. An Agilent ZORBAX Eclipse Plus C18 column, 2.1 mm × 100 mm, 3.5 μm was used for the chromatographic separations of uterine fluid samples with the column temperature maintained at 60 °C and the flow rate at 0.5 mL/min. Mobile phase A, consisting of 0.1% formic acid in water, and mobile phase B, consisting of 0.1% formic acid in acetonitrile, were used for gradient elution (see Table S4). The injected sample volume was 3 μL.

MS data acquisition was performed in both the positive and negative ion modes. The parameters used included source temperature (120 °C), desolvation temperature (500 °C), nitrogen gas flow rate (600 L/h), and cone gas flow rate (50 L/h). For the positive ion mode, the capillary, sampling cone, and extraction cone voltages were 3.0 kV, 27 eV, and 4 eV, respectively. For the negative ion mode, the capillary, sampling cone, and extraction cone voltages were 4.5 kV, 27 eV, and 4 eV, respectively. The full range mass scan was from 50~1500 *m*/*z*.

### 4.6. Identification of Metabolites and Statistical Analysis of Data

After obtaining the spectrogram of 18 uterine fluid samples through LC-MS, the raw mass spectrometry data were processed using Marker View software (Marker View 1.2.1, AB SCIEX, Redwood City, CA USA) to identify, filter, alignment, *m*/*z* value, and peak area were obtained by two-dimensional matrices and data were normalized by MetaboAnalyst 5.0 software. PLS-DA, volcano plot analysis (fold change ≥2 or ≤0.5, and *p* < 0.05), and variable importance in projection (VIP ≥ 1.0) analysis were performed using the MetaboAnalyst (https://www.metaboanalyst.ca/, accessed on 25 June 2020) online analysis platform and R project. Metabolites with FC≥2 or ≤0.5, *p* < 0.05, and VIP ≥1.0 were first annotated based on their exact mass data corresponding to *m*/*z* peaks by searching them against the Metlin and Human Metabolome Database databases to obtain their accession numbers, and then their names were identified by alignment to the PubChem database. Finally, the metabolites were subjected to - -KEGG- pathway enrichment analysis using the MBRole platform (http://csbg.cnb.csic.cs/mbrole, accessed on 28 June 2020). Combining statistical analysis and relevant literature on the functions of these differential metabolites, candidate metabolites affecting eggshell calcification were screened in our study.

### 4.7. Verification of Differential Metabolites Potentially Associated with Eggshell Quality

#### 4.7.1. Oral Administration

Among the potential metabolites related to eggshell calcification, two metabolites could only be deposited in chickens’ bodies through feed intake. Therefore, these two organics were fed to hens, and eggshell quality parameters were measured to verify whether the organics affected eggshell quality.

Two tablets of verapamil hydrochloride (40 mg/tablet, the Central Pharmaceutical Co., Ltd., Tianjin, China) and 3.5 tablets of sodium risedronate (5 mg/tablet, Kunming JiDa Pharmaceutical Co., Ltd., Kunming, China) were ground into powder, and then these powders were evenly filled in empty plant-based capsules; that is, an average of 8 mg of verapamil hydrochloride power or an average of 1.75 mg of sodium risedronate power was loaded into each capsule. Thirty 66-week-old White Leghorn hens (egg-laying rate >77%) kept on our experimental farm at China Agricultural University were randomly selected and divided into three groups (10 hens per group). After eggs were collected, hens of these three groups were fed verapamil hydrochloride capsules (1 capsule/hen, treatment group), sodium risedronate capsules (1 capsule/hen, treatment group), or empty capsules (1 capsule/hen, control group) at 11:00 am on the same day. Eggs produced by experimental hens on the day capsules were fed and on the successive 3 days after capsule administration were collected and used to measure eggshell strength, thickness, and weight. The eggshell quality traits were examined by one-way ANOVA using SAS 9.0 (SAS Institute, Cary, NC, USA).

#### 4.7.2. In Vitro Calcite Crystal Growth Assay

Two types of pigment molecules, CpgenIII and biliverdin, are considered to play an important role in improving eggshell quality. To better verify whether these molecules affect eggshell quality and understand how they affect the mechanical properties of eggshells, an in vitro calcite growth assay was developed to observe calcite crystal morphological changes and investigate the relationship between these two pigments and eggshell quality.

**Figure 6:**
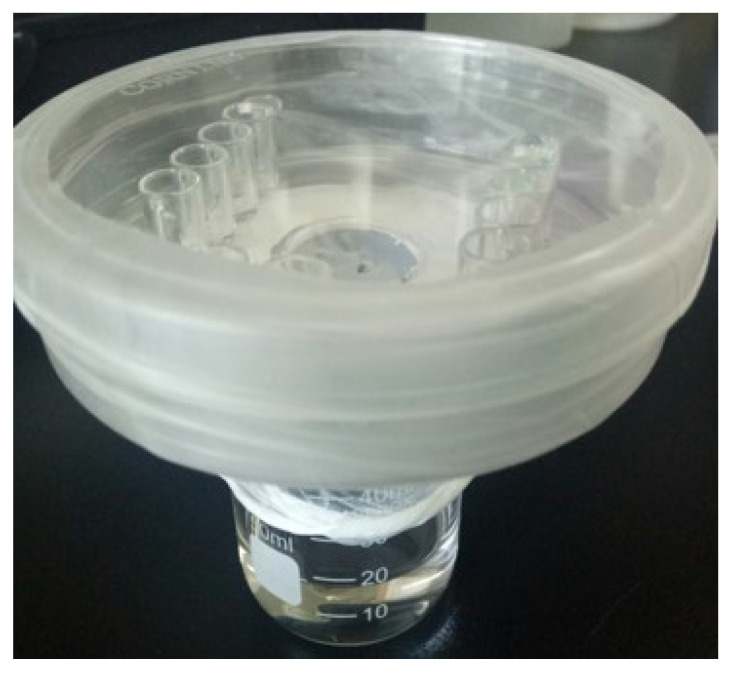
Experimental setup used for calcite crystal growth in vitro.

The crystallization method was performed by using gas diffusion according to a previous study [67] with some modifications (Figure 6). A small container was filled with 60 µL of CaCl_2_ solution (0.2 M) with or without either CpgenIII or biliverdin, and a single-side polishing silicon wafer <111> was totally immersed into CaCl_2_ solution. Every four small adjacent containers with the same solution were treated as an experimental group. Small containers were placed on a Petri dish with a center hole 2 cm in diameter on the bottom part. A 50-mL glass beaker containing 15 mL of ammonium carbonate solution (0.5 M) was covered by Parafilm with several pinholes for carbon dioxide diffusion. Then, the dish was placed over the glass beaker, and this closed system was sealed by thermal grease. The single-sided polished silicon wafers were collected after 2 h, rinsed with absolute ethanol and ultrapure water, dried at room temperature and then coated with gold, and the calcite crystal size and shape were observed by scanning electron microscopy.

## 5. Conclusions

For the first time, the current study screened the potential uterine fluid metabolites involved in eggshell calcification and eggshell quality with LC-MS-based metabolomics technology. Six crucial metabolites, phosphatidylcholine, diacylglycerol, verapamil, risedronate, coproporphyrinogen III, and biliverdin, were screened as potential matrixes affecting eggshell quality. According to a previous study and verification test, it can be concluded that high concentrations of uterine phosphatidylcholine are associated with higher eggshell strength. Verapamil decreased eggshell strength and thickness. The pigments coproporphyrinogen III and biliverdin could alter calcite crystal size and morphology and then strengthen eggshells. Our study provides a new perception of the function of uterine organic matrixes during eggshell calcification.

## Figures and Tables

**Table 1:** Comparison of eggshell quality traits of eggs laid by selected hens from the 3 groups.

	Eggs with Strong, Thick, Heavy Shells (3 eggs/hen, 6 hens)	Eggs with Weak, Thick, Heavy Shells (3 eggs/hen, 6 hens)	Eggs with Weak, Thin, Light Shells (3 eggs/hen, 6 hens)	Population (3 eggs/hen, 183 hens)
Eggshell strength (kg/cm^2^)	3.531 ± 0.045 ^a^	2.114 ± 0.141 ^c^	1.692 ± 0.473 ^c^	2.826 ± 0.585 ^b^
Eggshell thickness (mm)	0.327 ± 0.004 ^a^	0.314 ± 0.021 ^ab^	0.257 ± 0.020 ^c^	0.311 ± 0.027 ^b^
Eggshell weight (g)	6.12 ± 0.270 ^a^	5.78 ± 0.485 ^a^	4.73 ± 0.369 ^b^	5.87 ± 0.598 ^a^
Egg weight (g)	61.29 ± 3.201	59.51 ± 3.175	57.68 ± 3.593	61.29 ± 4.101
Shape index (length/width)	1.31 ± 0.016	1.29 ± 0.032	1.28 ± 0.004	1.30 ± 0.040

^a^^–c^ Mean values with no common superscripts within each row differ significantly (*p* < 0.05). All values are shown as the mean ± SD.

**Table 2:** KEGG pathway analysis of metabolites identified in positive ion mode ^1^.

Pathway	Raw p	KEGG Hits	Enriched Differential Metabolites
alpha-Linolenic acid metabolism	0.0385	C06427, C00157	PC(16:1(9Z)/22:6(4Z,7Z,10Z,13Z,16Z,19Z)), PC(15:0/P-16:0), PC(20:2(11Z,14Z)/P-18:1(11Z))
Glycerophospholipid metabolism	0.0445	C00157, C00350, C04230	PC(16:1(9Z)/22:6(4Z,7Z,10Z,13Z,16Z,19Z)), PC(15:0/P-16:0), PE(16:0/P-18:1(11Z)), PC(20:2(11Z,14Z)/P-18:1(11Z))

^1^ The metabolites detected in positive ion mode were not significantly enriched in certain KEGG pathways.

**Table 3:** Description of metabolite molecules potentially contributing to eggshell quality.

Metabolites	Molecular Weight	Comparison Group ^1^	Fold Change	*p* Value	Ion Mode ^2^
DG(14:0/20:3(5Z,8Z,11Z)/0:0)	591.4	STH, WTH	2.014	0.0032	+
DG(18:4(6Z,9Z,12Z,15Z)/24:1(15Z)/0:0)	699.5	STH, WtL	0.317	0.0004	+
PC(16:1(9Z)/22:6(4Z,7Z,10Z,13Z,16Z,19Z))	804.5	STH, WTH	0.497	0.0111	+, −
PC(15:0/P-16:0)	704.5	STH, WtL	0.275	0.0003	+, −
PC(20:2(11Z,14Z)/P-18:1(11Z))	796.6	WTH, WtL	2.516	2.79 × 10^−5^	+, −
Verapamil	453.3	WTH, WtL	4.167	0.0333	−
Risedronate	282.1	STH, WTH	0.472	0.0186	−
Coproporphyrinogen III	661.2	STH, WtL	3.088	0.0001	+
Biliverdin	583.2	STH, WtL	2.656	0.0001	+

^1^ STH, hens with strong, thick, and heavy eggshells; WTH, hens with weak, thick, and heavy eggshells; and WtL, hens with weak, thin, and light eggshells. ^2^ In the ion mode column, “+” indicates that the metabolites were only detected in positive mode, “−” means that the metabolites were only detected in negative mode, and “+, −” means the metabolites were detected both in the positive and negative modes.

**Table 4:** Diet composition and nutrition level of laying hens.

Ingredients	Content (%)	Nutritional Parameters	Levels
Soybean meal (43% CP)	24.56	AME (MJ/kg)	11.37
Corn	63.42	Crude protein (%)	17.28
Soybean oil	0.5	Calcium (%)	3.14
Coarse limestone	8.5	Total phosphorus (%)	0.33
Premix ^1^	3	l-Lysine (%)	0.886
Antioxidants	0.02	DL-Methionine (%)	0.281
Total	100		

^1^ Premix (provided per kilogram of feed) the following substances: vitamin A, 12,500 IU; vitamin D3, 2500 IU; vitamin K3, 2.65 mg; vitamin B1, 2 mg; vitamin B2, 6 mg; vitamin B12, 0.025 mg; vitamin E, 30 IU; biotin. 0.0325 mg; folic acid, 1.25 mg; pantothenic acid, 12 mg; niacin, 50 mg; Cu 8 mg; Zn, 75 mg; Fe 80 mg; Mn, 100 mg; Se, 0.15 mg, I, 0.35 mg.

## Data Availability

The data presented in this study are available in Supplementary Materials.

## Supplementary Materials

The following are available online at https://www.mdpi.com/article/10.3390/metabo11090575/s1, Table S1: Differential metabolites between the STH and WTH groups in positive and negative ion modes, Table S2: Differential metabolites between the STH and WtL groups in positive and negative ion modes, Table S3: Differential metabolites between the WTH and WtL groups in positive and negative ion modes, Table S4: The procedure of chromatographic gradient elution.
